# Avian leukosis virus (ALV) is highly prevalent in fancy-chicken flocks in Saxony

**DOI:** 10.1007/s00705-022-05404-y

**Published:** 2022-03-17

**Authors:** Markus Freick, Ruben Schreiter, Jim Weber, Thomas W. Vahlenkamp, Kristin Heenemann

**Affiliations:** 1 Faculty Agriculture/Environment/Chemistry, HTW Dresden-University of Applied Sciences, Pillnitzer Platz 2, 01326 Dresden, Germany; 2 ZAFT e.V.-Centre for Applied Research and Technology, Friedrich-List-Platz 1, 01069 Dresden, Germany; 3 Veterinary Practice Zettlitz, Straße der Jugend 68, 09306 Zettlitz OT Methau, Germany; 4 Veterinary Faculty, Center for Infectious Diseases, Institute of Virology, University of Leipzig, An den Tierkliniken 29, 04103 Leipzig, Germany

## Abstract

The current prevalence of avian leukosis virus (ALV) in fancy chickens in Germany is unknown. Therefore, 537 cloacal swabs from 50 purebred fancy-chicken flocks in Saxony were tested for the presence of the ALV p27 protein using a commercial antigen-capture ELISA. The detection rate was 28.7% at the individual-animal level and 56.0% at the flock level. Phylogenetic analysis of PCR products obtained from 22 different flocks revealed the highest similarity to ALV subtype K. When classifying breeds by their origin, ALV detection rates differed significantly. Evaluation of questionnaire data revealed no significant differences between ALV-positive and negative flocks regarding mortality.

**Supplementary Information:**

The online version contains supplementary material available at 10.1007/s00705-022-05404-y.

Avian leukosis virus (ALV), a member of the genus *Alpharetrovirus*, family *Retroviridae*, is the causal agent of avian leukemia [29]. The genome of ALV is approximately 7.2 kb in size and encodes structural and enzymatic proteins, such as gag, pol, and env. A major group-specific antigen is the capsid protein ALV p27, encoded by the gag gene [30].

Exogenous ALV induces neoplastic diseases of different pathotypes and productive disorders in chickens [7]. Moreover, this virus is known to cause subclinical infections in chickens and can be involved in conditions such as growth retardation, egg drop syndrome, and immunosuppression [21].

Breeding of fancy poultry is a popular leisure activity in Germany. In 2017, 286,739 pure-bred chickens in 26,952 fancy-chicken flocks were documented within in a stocktaking by the German Fancy-Poultry Breeders' Association. Within Germany, the states with the most fancy breeding chickens are Saxony and Thuringia [2]. Veterinary support in fancy-poultry breeding is poor, since it is not economically attractive for practitioners [9]. Thus, in general, little is known about the prevalence of diseases in fancy poultry or the significance of fancy poultry as a pathogen reservoir for commercial poultry farms [32]. Likewise, strategic programs for the control of ALV infections have not yet been implemented in fancy-chicken flocks in Germany, and data on the prevalence of ALV infection in purebred chickens is lacking. We therefore conducted this study to determine the detection rate of ALV in fancy-poultry flocks in Saxony. Furthermore, the effects of ALV infections and risk factors at the flock level were analyzed in a questionnaire-based survey.

This study was initiated by the Saxon Fancy-Poultry Breeders' Association (www.srv-gefluegel.de; Dresden, Germany) as a voluntary service offered to its members for infectious disease surveillance. In total, 537 cloacal swabs (sterile swabs without medium; WDT eG, Garbsen, Germany) from purebred chickens from 50 flocks in Saxony were collected by the breeders and submitted to the laboratory via courier in April 2016 (60 samples from five flocks) and from December 2016 to February 2017 (477 samples from 45 flocks). Data about individual birds (sex, age, and breed) were provided by the breeders on a sample submission form. Additionally, breeders were asked to fill out a questionnaire regarding the number of breeding animals, breeding flocks, chicks raised per year, mortality in adult chickens and chicks, data on chicken husbandry, biosecurity, and flock health management (Supplementary Table S1).

Samples were tested for the presence of ALV antigen using a commercial antigen-capture enzyme-linked immunosorbent assay (ELISA) (IDEXX ALV Ag test, Hoofdorp, The Netherlands) according to the manufacturer´s instructions. This ELISA detects ALV p27, an antigen common to all subgroups of ALV. The recommended sample types are light albumin or cloacal swabs [20], [33]. The results were expressed as relative optical density (OD) values, which were calculated as sample-to-positive ratio: S/P = (OD_sample_ – OD_negative control_)/(OD_positive control_ – OD_negative control_). Results of the ELISA were classified as positive (S/P >0.2), negative (S/P <0.2), or indeterminate (S/P = 0.2).

Samples collected in April 2016 were tested in the laboratory of the Institute of Poultry Diseases, Department of Veterinary Medicine, Free University of Berlin. All other samples were tested at the Institute of Virology, Veterinary Faculty, University of Leipzig.

DNA was isolated from cloacal swabs using a DNeasy Blood & Tissue Kit (QIAGEN, Hilden, Germany) according to manufacturer's instructions. A polymerase chain reaction (PCR) was performed as described by Smith et al. [25] for the detection of ALV provirus. For amplification of a portion of the polymerase gene, 2 µl of DNA was mixed with 5 µl of 5X Q5 Reaction Buffer (New England Biolabs, Ipswich, Massachusetts, USA), 0.5 µl of dNTPs (final concentration, 200 µM; Thermo Fisher Scientific, Waltham, Massachusetts, USA), 1.25 µl of forward primer ALV H5 and reverse primer AD1 (final concentration, 0.5 µM) and 0.25 µl of Q5 Hot Start High-Fidelity DNA Polymerase (final concentration, 0,02 U/µl) (New England Biolabs, Ipswich, MA, USA). The PCR protocol started with the activation of the polymerase for 30 s at 98 °C, followed by 40 cycles of denaturation for 10 s at 98 °C, annealing for 15 s at 60 °C, and elongation for 18 s at 72 °C. The reaction ended with a final elongation step for 2 min at 72 °C. Subsequently, the PCR product was analysed by agarose gel electrophoresis. Samples with a PCR product size between 295 and 326 base pairs were considered positive, and the PCR products were purified using a GeneJET PCR Purification Kit (Thermo Scientific, Germany) and sent to Microsynth Seqlab (Göttingen, Germany) for Sanger sequencing.

Nucleotide sequences were analyzed and edited using the GENtle program (Magnus Manske, University of Cologne, Germany) and compared to sequences in the National Center for Biotechnology Information (NCBI) database (https://www.ncbi.nlm.nih.gov/). Phylogenetic analysis and construction of phylogenetic trees was carried out by using the software MEGA X [10].

For collection and processing of data, a standard software package was used (Microsoft Office^®^, Microsoft Corporation, Redmond, USA). Individual animal data (sex, age, and breed) were extracted from the sample submission form. Breeds were categorized according to two different approaches. While the first approach considered body size of the breeds (three classes: large breeds, dwarf breeds, and original bantam breeds) in accordance with the German Fancy-Poultry Standard of Perfection [1], in the second model, breeds were classified regarding their origin on the basis of breeding history [1] and molecular genetic analysis [15, 24], 2015, [16] (five classes: breeds of Asian origin, North-Western European breeds/intermediate type breeds, Mediterranean breeds/East-European breeds, Gamecock and related breeds, and miscellaneous breeds) (Supplementary Table S2).

For statistical data analysis, the software SPSS^®^ Statistics version 25 (IBM^®^, Armonk, NY, USA) and WinSTAT^®^ (R. Fitch Software, Bad Krozingen/Germany) were used. The prevalence of ALV was calculated at the individual-animal level and at the flock level. A flock was considered ALV positive if at least one cloacal swab from the flock tested positive by ALV p27 ELISA. Confidence intervals for prevalence were estimated using the bootstrap method (n = 1000). For analyzing categorical data (i.e., ALV p27 ELISA results, ALV flock status, sex, age, breed, and nominal data obtained from the questionnaire), cross tables were created and the chi-squared test was used. If indicated, post-hoc pairwise comparison was conducted using Fisher´s exact test. If an event frequency in a cross table was lower than 5, Fisher´s exact test was applied. The Mann-Whitney U-test was used to compare continuous data (number of animals and mortality) between ALV-positive and negative flocks. Bonferroni correction was implemented to control for first-type error due to multiple testing. *P*-values ≤ 0.05 were considered statistically significant.

In total, 537 cloacal swabs from 50 purebred fancy-chicken flocks (Supplementary Table S2) from 44 breeders in Saxony were tested for the presence of ALV p27 protein by a commercial antigen-capture ELISA. On average, 10.8 ± 3.9 samples per flock (mean ± standard deviation) were submitted for laboratory examination. The detection rate (i.e., percentage of positive samples) was 28.7% (154/537) at the individual-animal level (95% CI: 24.2-31.9%) and 56.0% (28/50) at the flock level (95% CI: 42.0-70.0%) (Fig. 1). Three cloacal swabs (0.5%) were categorized as indeterminate. The distribution of flocks categorized as positive or negative in Saxony is shown in Supplementary Fig. S1. The mean proportion of cloacal swabs that tested positive for ALV within ALV-positive flocks was 46.5% (95% CI: 34.9-58.1%).

**Fig. 1:**
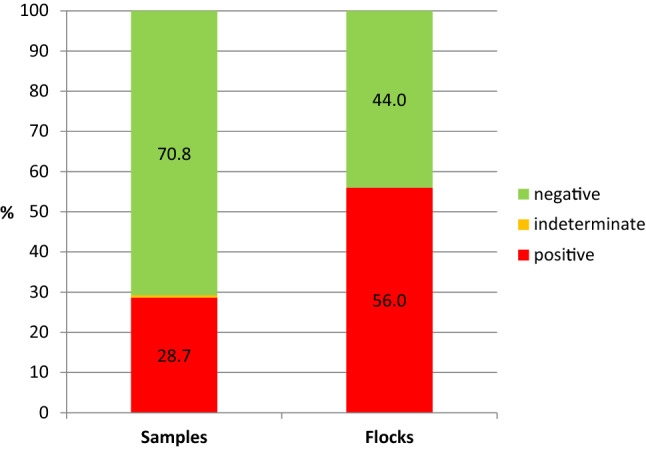
Detection rates of avian leukosis virus (ALV) determined using by a commercial ALV p27 ELISA with cloacal swabs from fancy chickens (n = 537, left bar) obtained from fancy-chicken flocks in Saxony (n = 50, right bar).

For further analysis of risk factors, positive and indeterminate samples were combined as "not negative" samples. No significant differences were observed in the ALV detection rate between roosters and hens (29.6 vs. 27.7%) and between the age categories (28.3 vs. 27.8%) (Table 1). When classifying breeds by their origin, ALV detection rates differed significantly at the individual-animal level between the categories, with a higher prevalence within the group of Mediterranean/East European breeds in comparison to Asian breeds, North-West European/intermediate type breeds, and gamecock breeds (Table 1, Fig. 2). However, this significance was not observed at the flock level (Supplementary Table S3). Evaluation of questionnaire data revealed no significant differences between ALV-positive and negative flocks regarding the number of chickens and mortality (Supplementary Table S4). Moreover, none of the analyzed factors regarding animal husbandry, biosecurity, and flock health management were significantly associated with ALV detection rates at the flock level (Supplementary Table S5).

**Table 1:** Distribution of avian leukosis virus (ALV) p27 ELISA results at the individual- animal level regarding sex, age, and breed categories

			ALV p27-ELISA		
Trait	Group	n samples (%)	n positive/indeterminate (%)	n negative (%)	*p*-value
Sex*	Male	115/527 (21.8)	34/115 (29.6)	81/115 (70.4)	0.689
	Female	412/527 (78.2)	114/412 (27.7)	298/412 (72.3)	
Age*	Young (≤1 year)	304/527 (57.7)	86/304 (28.3)	218/304 (71.7)	0.902
	Old (>1 year)	223/527 (42.3)	62/223 (27.8)	161/223 (72.2)	
Breed (body size)	Large breeds	247/537 (46.0)	73/247 (29.6)	174/247 (70.4)	0.947
	Dwarf breeds	197/537 (36.7)	56/197 (28.4)	141/197 (71.6)	
	Original bantam breeds	93/537 (17.3)	28/93 (30.1)	65/93 (69.9)	
Breed (origin)	Breeds of Asian origin	198/537 (36.9)	45/198 (22.7)	153/198 (77.3)	<0.001
	North-Western European breeds, intermediate type breeds	47/537 (8.8)	4/47 (8.5)	43/47 (91.5)	
	Mediterranean breeds, East-European breeds	79/537 (14.7)	49/79 (62.0)	30/79 (38.0)	
	Gamecock breeds and related	92/537 (17.1)	18/92 (19.6)	74/92 (80.4)	
	Miscellaneous (original bantam breeds, crested chicken breeds)	121/537 (22.5)	41/121 (33.9)	80/121 (66.1)	

^*^ 10 samples from chickens of unknown sex and age

**Fig. 2:**
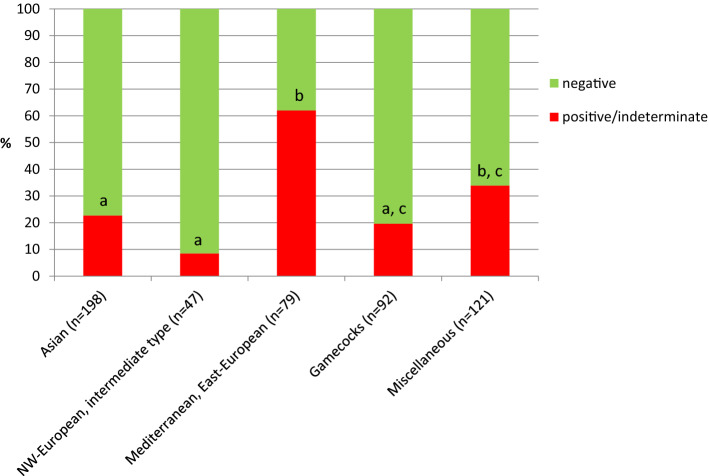
Detection rates of avian leukosis virus (ALV) determined using a commercial ALV p27 ELISA with cloacal swabs from fancy chickens (n = 537) categorized according to the origin of the breed. Different indices indicate significant differences between the bars (*p* ≤ 0.05).

From each positive flock (n = 28), one sample was selected randomly for analysis by PCR. If there was more than one positive sample in the flock, one sample was selected by generating random numbers in Microsoft Excel (Microsoft Office^®^, Microsoft Corporation, Redmond, USA). All selected samples showed a positive PCR result. The PCR product was successfully sequenced from 22 of the samples examined, and the sequences were submitted to the NCBI database (accession nos. MZ504880 to MZ504901, Supplementary Table S6). Avian leukosis virus strain TW-3593, new subgroup K (NCBI accession no. HM582658, protein ID ADP21278.1), was selected as a reference strain. The ALV isolates analyzed in this study showed 98.89 to 100% sequence identity at the nucleotide (nt) level and 98.04 to 100% identity at the amino acid (aa) level to the reference strain (Supplementary Table S6). Phylogenetic analysis of ALV reference sequences and the partial polymerase gene sequences from this study (Supplementary Fig. S2) further support the inclusion of the strains detected in Saxony in ALV subgroup K.

This study provides current data on the distribution of ALV in fancy-chicken flocks in Saxony. The high detection rates at the individual-animal and flock level is in contrast to the situation in the commercial poultry industry, where ALV-A, B, C, D, and J have been eliminated from the breeding flocks of most international breeding companies by strict eradication programs [23, 34]. However, there has been evidence in recent years for the sustained presence of exogenous ALV in Asian native chicken breeds [8, 11, 18, 19, 26, 34, 35] as well as in chickens in European countries, including Greece [31] and Switzerland [32]. This difference in ALV prevalence between commercial breeding flocks and fancy chickens might be explained by the lack of implementation of programs to control ALV infections in fancy and backyard chickens and their significant lower biosecurity level, which offers opportunities for viral spread between flocks and over generations within a flock. In fancy-chicken flocks, different breeds of various ages are commonly kept together (i.e., multi-age flocks), and birds and/or hatching eggs are traded intensively to improve the breeding stock. In addition, regular visits to local, regional, or international exhibitions are common. The implementation of preventive measures such as hygiene concepts and disease monitoring is unusual [32].

ALV can be transmitted horizontally and vertically, but congenital transmission via hatching eggs is more important for tumorous leukosis outbreaks [17]. The critical roles of infected hens in transmission of ALV from generation to generation on chicken farms have been emphasized previously [17, 21]. In contrast, the role of roosters in the transmission of ALV is not fully understood. However, a recent study indicated that females that were horizontally infected late by ALV-J-infected semen might transmit the virus to their progeny through their eggs, which amounts to vertical transmission of the virus [14]. The transmission routes described above may explain the high proportion of ALV-positive cloacal swabs within ALV-infected flocks detected in this study. In fancy-chicken breeding, hens and roosters with high breeding value are used extensively to produce a large number of offspring. Thus, an ALV-infected breeding bird can contribute to widespread transmission of the virus within the flock, particularly because many fancy-chicken flocks are small.

Notably, ALV prevalence differed significantly between breeds classified based on their origin and breeding history. This may indicate differences in ALV susceptibility between the breeds. Resistance to infection by a particular ALV subgroup can be caused by genetic alterations in specific receptor genes. Resistant alleles (i.e., *tva*^*r*^, *tvb*^*r*^, *tvc*^*r*^, and *tvj*^*r*^) have been identified in all four receptor loci [4]. Against this background, purebred fancy chickens could be promising target animals in the search for additional resistance genes.

Phylogenetic analysis of PCR products from our study revealed the highest nucleotide sequence similarity to ALV-K strains, indicating the presence of only one ALV subtype in fancy-chicken flocks in Saxony. Likewise, a complex pattern of ALV infection has been reported in native chicken breeds in China [12]. Recently, several ALV strains isolated from indigenous chicken breeds in China, Taiwan, and Japan were proposed to represent a new subgroup of ALV, designated as ALV-K, on the basis of amino acid sequence similarity in the gp 85 envelope protein [3, 5, 6, 28]. Li et al. [2016] demonstrated that ALV-K strain GD14LZ replicated more slowly in DF-1 cells than the GD08 (ALV-A) and NX0101 (ALV-J) strains and did not induce tumor formation in specific-pathogen-free (SPF) chickens. Since ALV-K is widespread in Saxony, this might explain the lack of increased mortality in ALV-infected flocks in this study. However, in the future, comprehensive phylogenetic studies of a larger number of local ALV isolates are necessary to determine the prevalence of ALV subgroups, possible recombination events [13, 27], and their importance for animal health in fancy chickens in Germany. Mutations in the polymerase gene have resulted in alteration of biological characteristics in ALV-K in China, which coincides with higher replication efficiency [22]. Unfortunately, the region of the polymerase gene where these unique mutations occurred was not included in the region amplified in this study und should be investigated in future studies.

For our investigations, samples were submitted by the breeders on a voluntary basis (convenience sampling). This suggests a risk of bias, as no random sampling was performed, and thus, some types of flocks may be overrepresented (e.g., flocks from breeders with a special interest in health management, or flocks with health problems). Additionally, only fancy-chicken flocks in Saxony were investigated. Therefore, our results may not reflect the situation in other federal states of Germany or the country as a whole. However, since the structures and organization in fancy-poultry breeding are very similar in the other German federal states, similar findings are likely. In this study, the sample size at the flock level was limited, which reduced the statistical power for identifying potential risk factors for ALV infections. Considering these limitations, further investigations including a supra-regional study design, random sampling strategies, and a larger sample size are proposed. In particular, traits factors associated with large differences in ALV detection rates for which there is a consistent biological explanation (e.g., purchase of hatching eggs) appear worthy of further consideration as potential risk factors for introduction of ALV into flocks.

In conclusion, we have demonstrated that the presence of ALV is widespread in fancy-chicken flocks in Saxony, with a high proportion of fecal shedders of ALV in ALV-positive flocks. Therefore, fancy chickens should be considered a potential reservoir for ALV. High priority should be given to biosecurity by poultry farmers and veterinarians to prevent of introduction the virus on commercial poultry farms, especially because of the recent increase in free-range management in commercial egg and poultry meat production [32]. Significant differences in ALV detection rates between breeds were observed at the individual-animal level. Thus, purebred fancy poultry might represent a promising target for detection of resistance genes, which could be beneficial if preserved and introduced into commercial hybrid lines.

## Supplementary Information

Below is the link to the electronic supplementary material.

**Supplementary Table S3** Distribution of avian leukosis virus (ALV) flock status regarding breeds classified according to body size or origin. A flock was categorized as ALV positive if at least one cloacal swab from the flock tested positive by ALV p27 ELISA. **Supplementary Table S4** Analysis of questionnaire data on flock size and mortality in relation to avian leukosis virus (ALV) flock status. A flock was categorized as ALV positive if at least one cloacal swab from the flock tested positive by ALV p27 ELISA. **Supplementary Table S5** Analysis of questionnaire data on chicken husbandry, biosecurity, and health management in relation to avian leukosis virus (ALV) flock status. A flock was categorized as ALV positive if at least one cloacal swab from the flock tested positive by ALV p27 ELISA. **Supplementary Table S6** Phylogenetic analysis of PCR products obtained from 22 different flocks revealed the closest similarity to ALV-K. Presentation of NCBI accession no., country, state, region, host (breed), collection date, and analysis of identity level compared to the reference strain NCBI accession no. HM582658/protein ID ADP21278.1 based on nucleotides (nt) and on amino acids (aa) (DOCX 77 KB)

**Supplementary Fig. S1** Location of the fancy-chicken flocks in Saxony (n = 50) from which cloacal swabs (n = 537) were collected to detect avian leukosis virus (ALV) using a commercial ALV p27 ELISA. Red dots, flocks tested positive for ALV; green dots, flocks tested negative for ALV. The map was created using batchgeo.com software (PPTX 350 KB)

Supplementary Fig. S2 Phylogenetic analysis based on complete genome (a) and partial polymerase gene sequences (b). The phylogenetic trees were constructed using MEGA X software, by the neighbor-joining method based on nucleotides (nt). The evolutionary distances were calculated using the Jukes-Cantor method with 1000 bootstrap replicates. The unrooted trees have a site coverage cutoff of 95%. (a) The phylogenetic analysis involved 11 reference sequences from NCBI, five sequences representing subgroup K (KU605774, HM582658, KP686142, KY773911 and KF746200, indicated by a triangle), and one sequence from each subgroup (ALV-A: L10922; ALV-B: AF052428, ALV-C: J02342, ALV-D: D10652, ALV-E: AY013303). There were a total of 6985 positions in the final dataset. (b) Partial polymerase gene sequences (261 nt, shorter sequences were excluded) were analyzed in comparison to sequences of 11 reference gene sequences from NCBI. The analysis involved 27 nucleotide sequences. The samples belonging to this study are indicated by a black square; reference sequences of ALV subgroup K are indicated by a triangle. (PNG 34 KB)

Supplementary file4 (PNG 34 KB)

Supplementary Table S2 List of 36 fancy chicken breeds from 50 flocks included in this study and their assignment to classes according to body size (classification A) and origin of the breed (classification B) (XLSX 12 KB)

Supplementary file6 (PPTX 125 KB)

Supplementary Table S1 Questionnaire on flock data provided to the breeders participating in this study (DOCX 103 KB)

## Data Availability

The datasets generated during and/or analysed during the current study are available from the corresponding author on reasonable request.
