# Supplementary material for: Avian leukosis virus (ALV) is highly prevalent in fancy-chicken flocks in Saxony
Source: Arch Virol. 2022 Mar 17;167(4):1169–74. doi: 10.1007/s00705-022-05404-y (PMC8964621; doi:10.1007/s00705-022-05404-y)
Supplement: Supplementary file 1 — Supplementary Table S3 Distribution of avian leukosis virus (ALV) flock status regarding breeds classified according to body size or origin. A flock was categorized as ALV positive if at least one cloacal swab from the flock tested positive by ALV p27 ELISA. Supplementary Table S4 Analysis of questionnaire data on flock size and mortality in relation to avian leukosis virus (ALV) flock status. A flock was categorized as ALV positive if at least one cloacal swab from the flock tested positive by ALV p27 ELISA. Supplementary Table S5 Analysis of questionnaire data on chicken husbandry, biosecurity, and health management in relation to avian leukosis virus (ALV) flock status. A flock was categorized as ALV positive if at least one cloacal swab from the flock tested positive by ALV p27 ELISA. Supplementary Table S6 Phylogenetic analysis of PCR products obtained from 22 different flocks revealed the closest similarity to ALV-K. Presentation of NCBI accession no., country, state, region, host (breed), collection date, and analysis of identity level compared to the reference strain NCBI accession no. HM582658/protein ID ADP21278.1 based on nucleotides (nt) and on amino acids (aa) (DOCX 77 KB) [file 705_2022_5404_MOESM1_ESM.docx]

**Supplementary Table S3**

|  |  |  | ALV flock status | |  |
| --- | --- | --- | --- | --- | --- |
| Trait | Group | n flocks (%) | n positive (%) | n negative (%) | *p*-value |
| Breed (body size) | Large breeds | 22/50 (44.0) | 13/22 (59.1) | 9/22 (40.9) | 0.773 |
|  | Dwarf breeds | 20/50 (40.0) | 10/20 (50.0) | 10/20 (50.0) |  |
|  | Original bantam breeds | 8/50 (16.0) | 5/8 (62.5) | 3/8 (37.5) |  |
| Breed (origin) | Breeds of Asian origin | 20/50 (40.0) | 9/20 (45.0) | 11/50 (55.0) | 0.195 |
|  | North-Western European breeds, intermediate type breeds | 6/50 (12.0) | 3/6 (50.0) | 3/6 (50.0) |  |
|  | Mediterranean breeds, East-European breeds | 5/50 (10.0) | 5/5 (100.0) | 0/5 (0.0) |  |
|  | Gamecock breeds and related | 7/50 (14.0) | 3/7 (42.9) | 4/7 (57.1) |  |
|  | Miscellaneous  (original bantam breeds, crested chicken breeds) | 12/50 (24.0) | 8/12 (66.7) | 4/12 (33.3) |  |

**Supplementary Table S4**

| Trait |  | ALV flock status* | |  |
| --- | --- | --- | --- | --- |
| Attribute | Unit | Negative | Positive | *p*-value |
| Number of breeding flocks | n (mean ± SD) | 5.1 ± 4.9 | 5.5 ± 4.8 | 0.977 |
| Number of breeding birds | n (mean ± SD) | 24.5 ± 23.0 | 27.9 ± 20.2 | 0.297 |
| Number of chicks (per year) | n (mean ± SD) | 92.1 ± 67.5 | 103.1 ± 92.1 | 0.824 |
| Mortality chicks (during rearing period) | % (mean ± SD) | 14.3 ± 26.1 | 4.8 ± 8.7 | 0.188 |
| Mortality adults (per year) | % (mean ± SD) | 1.1 ± 0.2 | 1.1 ± 0.3 | 0.673 |

* flocks: n = 50; missing questionnaire data for one flock

**Supplementary Table S5**

| Trait | Class | n ALV positive flocks* (%) | *p*-value |
| --- | --- | --- | --- |
| Type of breeding facility | individual | 30/46 (65.2) | 1.000 |
|  | collective | 2/3 (66.6) |  |
| Hatching | individual | 23/35 (65.7) | 1.000 |
|  | collective | 9/14 (64.3) |  |
| Rearing facility | not separated from breeding facility | 30/46 (65.2) | 1.000 |
|  | separated from  breeding facility | 2/3 (66.6) |  |
| Routine laboratory diagnostics in breeding flock | no | 21/30 (70.0) | 0.539 |
|  | yes | 11/19 (57.9) |  |
| Attendance at poultry exhibitions | no | 0/1 (0.0) | 0.346 |
|  | yes | 32/48 (66.7) |  |
| Periodic purchase of breeding birds | no | 13/20 (65.2) | 1.000 |
|  | yes | 19/29 (65.5) |  |
| Periodic purchase of hatching eggs | no | 19/33 (57.6) | 0.123 |
|  | yes | 13/16 (81.3) |  |
| Contact of fancy chickens with commercial hybrid chickens | no | 30/45 (66.7) | 0.602 |
|  | yes | 2/4 (50.0) |  |
| Contact of fancy chickens with other poultry species | no | 17/23 (73.9) | 0.367 |
|  | yes | 15/26 (57.7) |  |
| Vaccination against Marek´s disease** | no | 6/13 (46.2) | 0.090 |
|  | yes | 26/35 (74.3) |  |
| Formerly animals within flock diagnosed positive for ALV** | no | 28/43 (65.1) | 0.652 |
|  | yes | 4/5 (80.0) |  |

* flocks: n = 50; missing questionnaire data for one flock ** according to the owner´s data

**Supplementary Table S6**

| NCBI Accession No. | Country: State, Region | Host (breed) | Collection date | Reference strain TW3593 China  (NCBI Acc No. HM582658/  protein ID ADP21278.1) | | |
| --- | --- | --- | --- | --- | --- | --- |
|  |  |  |  | position nt | % ident nt | % ident aa |
| MZ504880 | Germany: Saxony, Zschopau,  OT Krumhermersdorf | Gallus gallus domesticus (Italian) | Dec-2016 | 5209-5472 | 99.62 | 100.00 |
| MZ504881 | Germany: Saxony, Dresden | Gallus gallus domesticus (Cochin bantam) | Dec-2016 | 5209-5478 | 99.26 | 98.04 |
| MZ504882 | Germany: Saxony, Schoenau-Berzdorf | Gallus gallus domesticus (Malay) | Dec-2016 | 5209-5457 | 99.20 | 100.00 |
| MZ504883 | Germany: Saxony, Neustadt, District Saechsische Schweiz-Osterzgebirge | Gallus gallus domesticus (Cornish Game bantam) | Jan-2017 | 5209-5478 | 99.63 | 100.00 |
| MZ504884 | Germany: Saxony, Community Wechselburg | Gallus gallus domesticus (Amrock bantam) | Jan-2017 | 5209-5478 | 99.26 | 98.04 |
| MZ504885 | Germany: Saxony, Community Duerrroehrsdorf-Dittersbach, OT Dobra | Gallus gallus domesticus (German Wyandotte bantam) | Jan-2017 | 5209-5471 | 99.62 | 100.00 |
| MZ504886 | Germany: Saxony, Kirchbach (Oederan) | Gallus gallus domesticus (German Wyandotte bantam) | Jan-2017 | 5209-5478 | 99.26 | 98.04 |
| MZ504887 | Germany: Saxony, Oederan | Gallus gallus domesticus (Rosecomb bantam) | Jan-2017 | 5209-5478 | 100.00 | 100.00 |
| MZ504888 | Germany: Saxony, Koenigswalde, Erzgebirgskreis | Gallus gallus domesticus (Niederrheiner) | Jan-2017 | 5209-5478 | 99.26 | 98.04 |
| MZ504889 | Germany: Saxony, Gruenhain-Beierfeld, Erzgebirgskreis | Gallus gallus domesticus (Silkie) | Jan-2017 | 5209-5478 | 98.89 | 98.04 |
| MZ504890 | Germany: Saxony, Luebau, Rabenau | Gallus gallus domesticus (Silkie) | Jan-2017 | 5209-5478 | 99.63 | 100.00 |
| MZ504891 | Germany: Saxony, Gruenhain- Beierfeld, Erzgebirgskreis | Gallus gallus domesticus (Booted bantam) | Jan-2017 | 5209-5472 | 99.62 | 100.00 |
| MZ504892 | Germany: Saxony, Neukirchen, Erzgebirgskreis | Gallus gallus domesticus (Italian) | Jan-2017 | 5221-5451 | 100.00 | 100.00 |
| MZ504893 | Germany: Saxony, Seustadt/Sa, Community Saechsische Schweiz-Osterzgebirge | Gallus gallus domesticus (Welsume) | Jan-2017 | 5209-5451 | 100.00 | 100.00 |
| MZ504894 | Germany: Saxony, Dippoldiswalde, Community Saechsische Schweiz-Osterzgebirge | Gallus gallus domesticus (Naked Neck) | Jan-2017 | 5209-5468 | 99.62 | 100.00 |
| MZ504895 | Germany: Saxony, Gruenhain-Beierfeld, Erzgebirgskreis | Gallus gallus domesticus (Italian) | Jan-2017 | 5209-5472 | 99.62 | 100.00 |
| MZ504896 | Germany: Saxony, Oberschoena, Community Mittelsachsen | Gallus gallus domesticus (Italian) | Jan-2017 | 5209-5471 | 99.62 | 100.00 |
| MZ504897 | Germany: Saxony, Zwoenitz, Erzgebirgskreis | Gallus gallus domesticus (Booted bantam) | Jan-2017 | 5209-5478 | 99.63 | 100.00 |
| MZ504898 | Germany: Saxony, Burkhardtsdorf, Community Gornsdorf, Erzgebirgskreis | Gallus gallus domesticus (Booted bantam) | Jan-2017 | 5209-5472 | 99.62 | 100.00 |
| MZ504899 | Germany: Saxony, Zwoenitz, Erzgebirgskreis | Gallus gallus domesticus (Booted bantam) | Jan-2017 | 5221-5440 | 99.09 | 100.00 |
| MZ504900 | Germany: Saxony, Oederan | Gallus gallus domesticus (Dutch bantam) | Feb-2017 | 5209-5453 | 99.18 | 100.00 |
| MZ504901 | Germany: Saxony, Zschopau, OT Krumhermersdorf | Gallus gallus domesticus (Italian) | Feb-2017 | 5209-5462 | 99.21 | 98.04 |
