# Supplementary figures and images for: Avian leukosis virus (ALV) is highly prevalent in fancy-chicken flocks in Saxony
Source: Arch Virol. 2022 Mar 17;167(4):1169–74. doi: 10.1007/s00705-022-05404-y (PMC8964621; doi:10.1007/s00705-022-05404-y)

## Slide 1
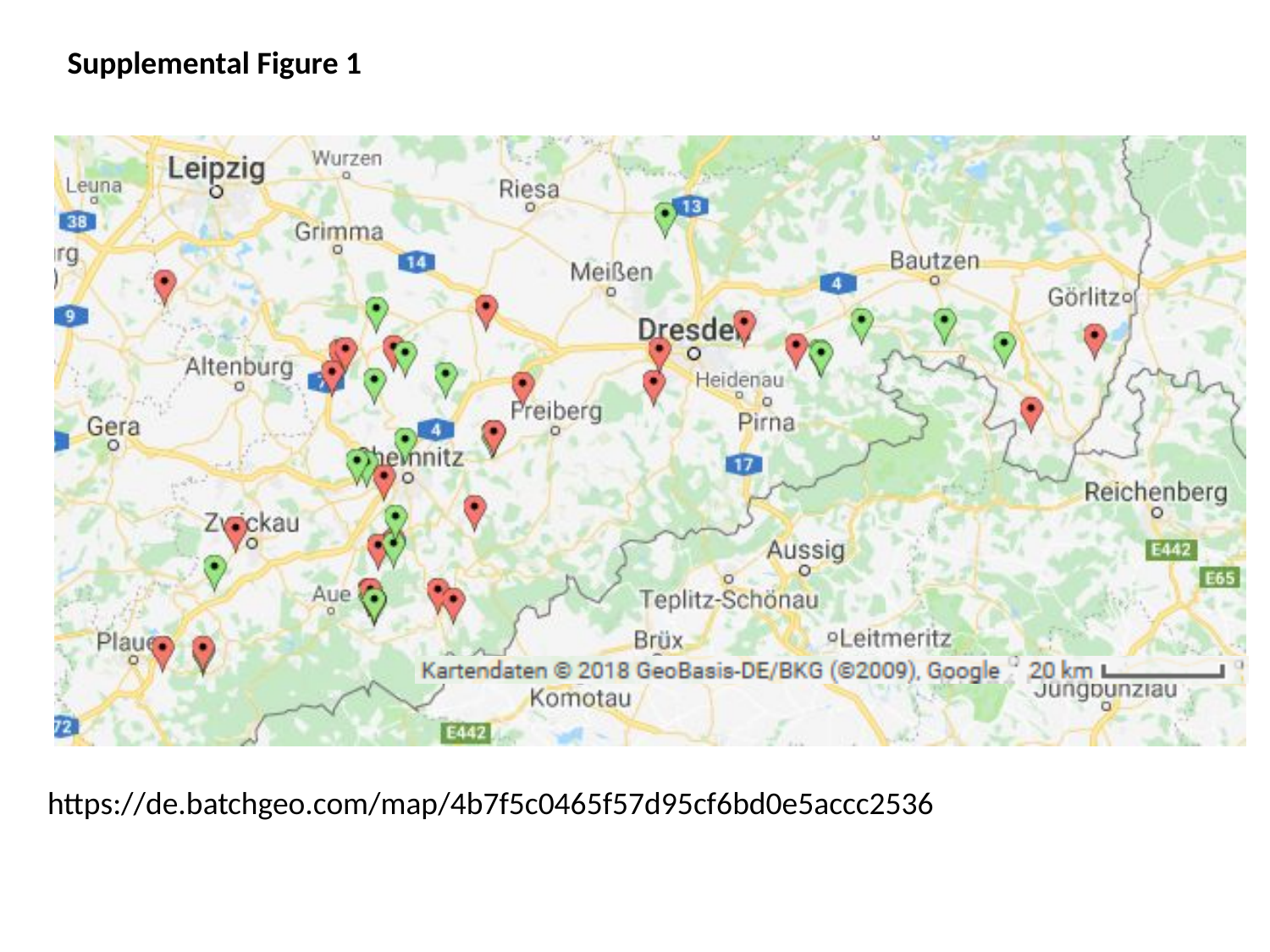

Supplemental Figure 1
https://de.batchgeo.com/map/4b7f5c0465f57d95cf6bd0e5accc2536

Supplement: Supplementary file 2 — Supplementary Fig. S1 Location of the fancy-chicken flocks in Saxony (n = 50) from which cloacal swabs (n = 537) were collected to detect avian leukosis virus (ALV) using a commercial ALV p27 ELISA. Red dots, flocks tested positive for ALV; green dots, flocks tested negative for ALV. The map was created using batchgeo.com software (PPTX 350 KB) [file 705_2022_5404_MOESM2_ESM.pptx]

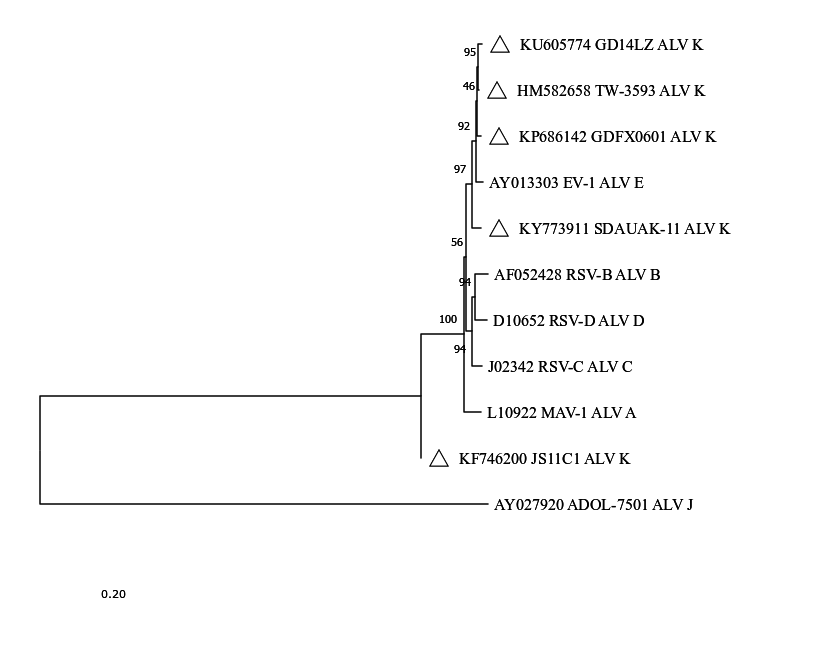

Supplement: Supplementary file 3 — Supplementary Fig. S2 Phylogenetic analysis based on complete genome (a) and partial polymerase gene sequences (b). The phylogenetic trees were constructed using MEGA X software, by the neighbor-joining method based on nucleotides (nt). The evolutionary distances were calculated using the Jukes-Cantor method with 1000 bootstrap replicates. The unrooted trees have a site coverage cutoff of 95%. (a) The phylogenetic analysis involved 11 reference sequences from NCBI, five sequences representing subgroup K (KU605774, HM582658, KP686142, KY773911 and KF746200, indicated by a triangle), and one sequence from each subgroup (ALV-A: L10922; ALV-B: AF052428, ALV-C: J02342, ALV-D: D10652, ALV-E: AY013303). There were a total of 6985 positions in the final dataset. (b) Partial polymerase gene sequences (261 nt, shorter sequences were excluded) were analyzed in comparison to sequences of 11 reference gene sequences from NCBI. The analysis involved 27 nucleotide sequences. The samples belonging to this study are indicated by a black square; reference sequences of ALV subgroup K are indicated by a triangle. (PNG 34 KB) [file 705_2022_5404_MOESM3_ESM.png]

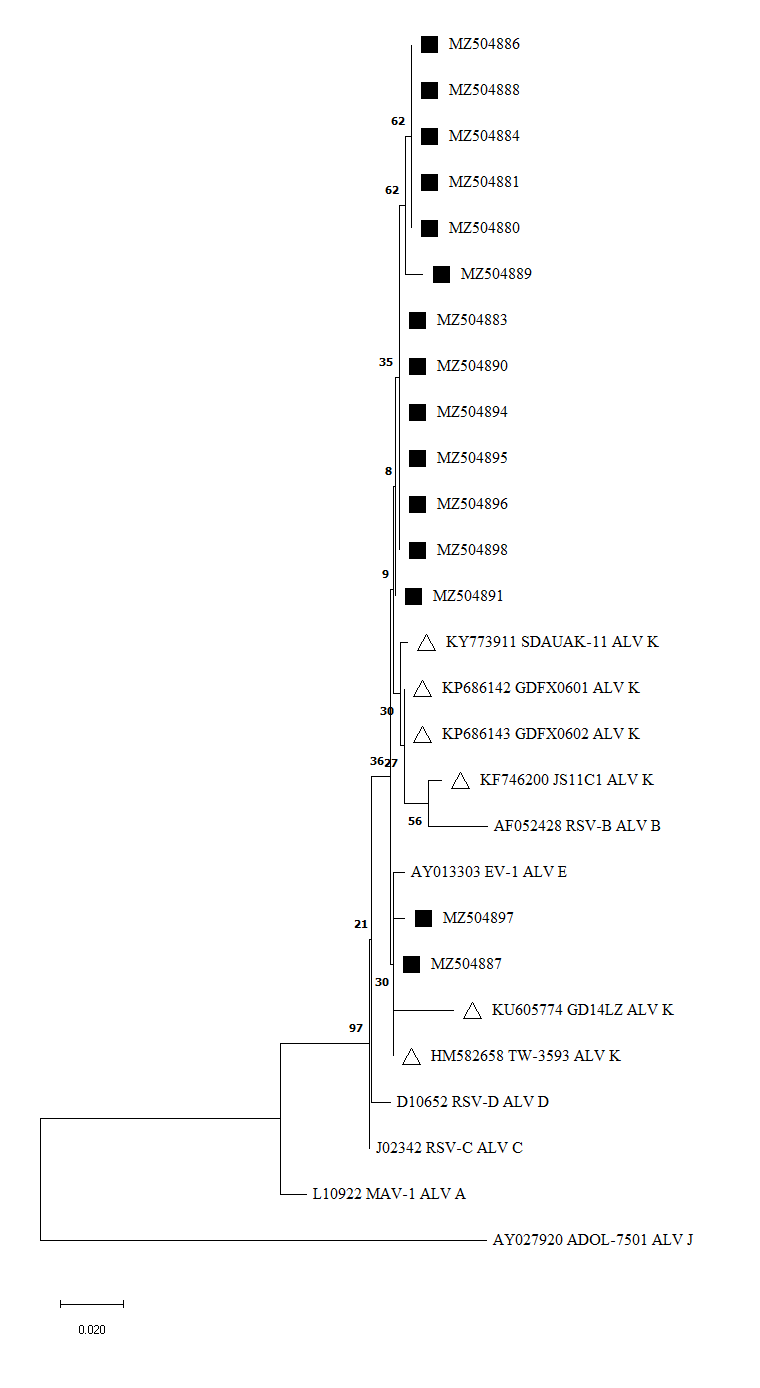

Supplement: Supplementary file 4 — Supplementary file4 (PNG 34 KB) [file 705_2022_5404_MOESM4_ESM.png]
