# Supplementary material for: Avian leukosis virus (ALV) is highly prevalent in fancy-chicken flocks in Saxony
Source: Arch Virol. 2022 Mar 17;167(4):1169–74. doi: 10.1007/s00705-022-05404-y (PMC8964621; doi:10.1007/s00705-022-05404-y)
Supplement: Supplementary file 6 — Supplementary file6 (PPTX 125 KB) [file 705_2022_5404_MOESM6_ESM.pptx]

## Slide 1
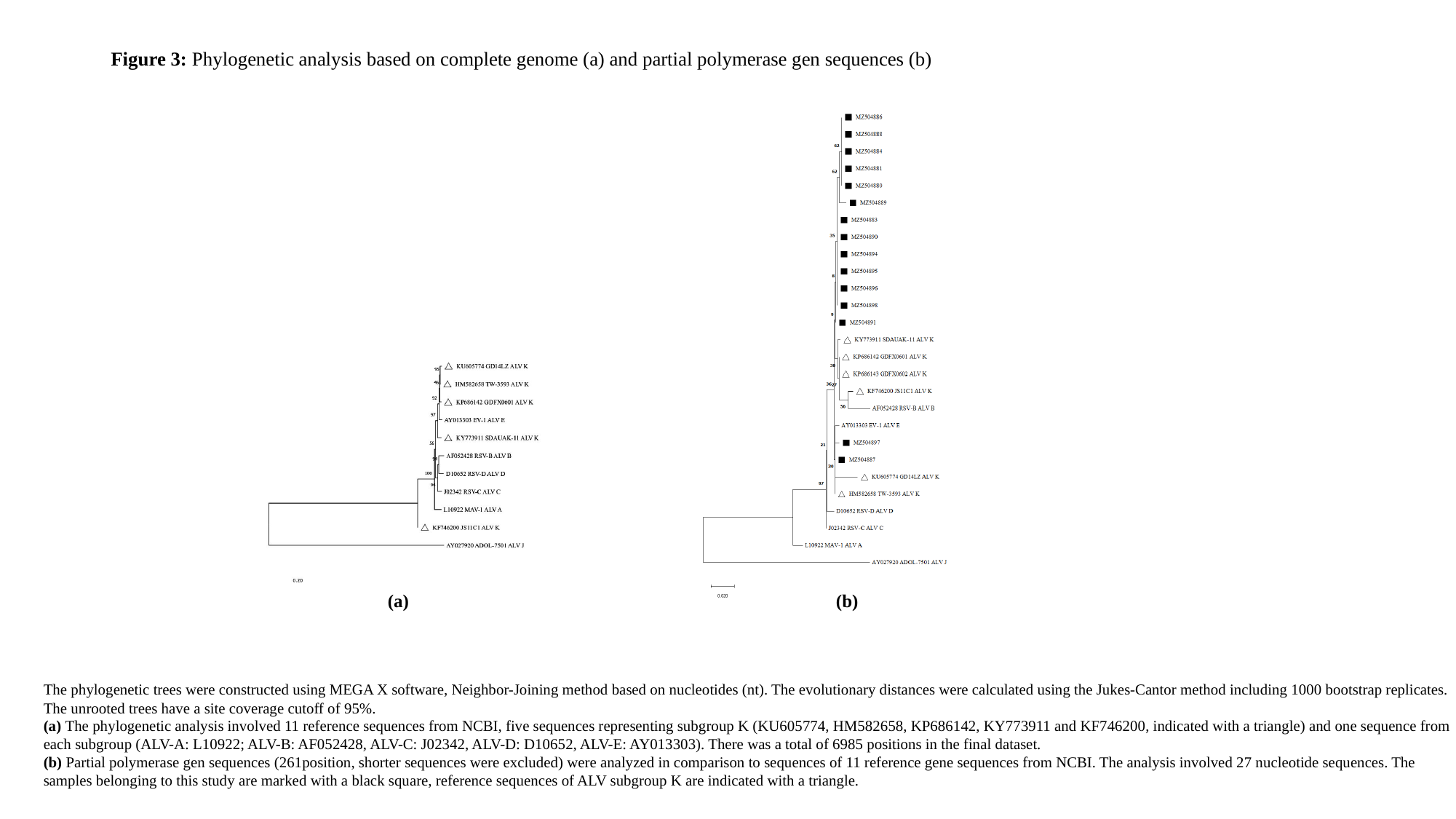

# Figure 3: Phylogenetic analysis based on complete genome (a) and partial polymerase gen sequences (b)
(a)
(b)
The phylogenetic trees were constructed using MEGA X software, Neighbor-Joining method based on nucleotides (nt). The evolutionary distances were calculated using the Jukes-Cantor method including 1000 bootstrap replicates. The unrooted trees have a site coverage cutoff of 95%.
(a) The phylogenetic analysis involved 11 reference sequences from NCBI, five sequences representing subgroup K (KU605774, HM582658, KP686142, KY773911 and KF746200, indicated with a triangle) and one sequence from each subgroup (ALV-A: L10922; ALV-B: AF052428, ALV-C: J02342, ALV-D: D10652, ALV-E: AY013303). There was a total of 6985 positions in the final dataset.
(b) Partial polymerase gen sequences (261position, shorter sequences were excluded) were analyzed in comparison to sequences of 11 reference gene sequences from NCBI. The analysis involved 27 nucleotide sequences. The samples belonging to this study are marked with a black square, reference sequences of ALV subgroup K are indicated with a triangle.
