# Supplementary material for: Avian leukosis virus (ALV) is highly prevalent in fancy-chicken flocks in Saxony
Source: Arch Virol. 2022 Mar 17;167(4):1169–74. doi: 10.1007/s00705-022-05404-y (PMC8964621; doi:10.1007/s00705-022-05404-y)
Supplement: Supplementary file 7 — Supplementary Table S1 Questionnaire on flock data provided to the breeders participating in this study (DOCX 103 KB) [file 705_2022_5404_MOESM7_ESM.docx]

# **Saxonian Fancy Poultry Breeder´s Association – Questionnaire for Avian Leukosis Project**


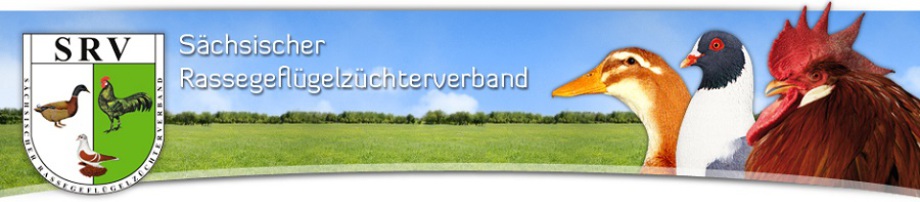


| Breeder |  | Name |  |
| --- | --- | --- | --- |
|  |  | Address |  |
|  |  | Club |  |
|  |  | District |  |

Please fill in or mark with a cross. For sampled animals, please record age and sex.

1. **Name of breed(s)**
2. **Number of breeding animals**

Roosters:

Hens:

Breeding groups:

1. **Breeding facility**

Single facility

Club breeding facility

1. **Number of hatched chicks per year (average)**
2. **Hatching**

Own icubator

Club hatchery

Commercial hatchery

1. **Is the rearing stable separated from the breeding animal´s pen?**

no

yes

if yes, distance (km):

1. **Mortality during the last rearing season (%):**
2. **Regularly disease-related mortality in breeding animals during the last year?**

no

yes

1. **Is a diagnostic clarification of animal losses implemented in the herd management (e.g., necropsy, swabs, fecal samples, blood samples)?**

no

yes, routinely

yes, sporadically

1. **Participation in poultry exhibitions?**

no

yes, only local (district)

yes, national/international

1. **Periodic purchase/exchange of breeding animals?**

no

yes

if yes, when was the last purchase/exchange (year):

1. **Periodic purchase/exchange of hatching eggs?**

no

yes

if yes, when was the last purchase/exchange (year):

1. **Are the fancy chickens kept together with commercial hybrid chickens (laying hens or broilers)?**

no

yes

1. **Are the fancy chickens kept together with other poutry species?**

no

yes

if yes, which species:

1. **Vaccinations in the fancy chicken herd**

Newcastle Disease

Marek´s Disease

Coccidiosis

Infectious Bronchitis

Infectious Laryngotracheitis

Other:

1. **Has avian leukosis already been diagnosed in the flock?**

no

yes

if yes, when (year):
